# Multivessel disease in STEMI patients: a perspective from limited-resource settings

**DOI:** 10.5830/CVJA-2018-034

**Published:** 2018

**Authors:** Ahmed Suliman Ahmed Ali, Naseer Nauman, Gersh Bernard

**Affiliations:** University of Khartoum, Khartoum, Sudan; Department of Cardiology, Akhtar Saeed Medical College, Lahore, Pakistan; Department of Medicine, Mayo Clinic, College of Medicine, Rochester, Minnesota, USA

**Keywords:** debate, Africa STEMI Live, multivessel disease, STEMI, limited-resource setting, infarct-related artery, noninfarct- related artery, complete revascularisation

During the Africa STEMI Live meeting held in Nairobi from 26 to 28 of April 2018, the Great Debate session was on how to approach non-infarct-related (N-IRA) disease in patients who present with STEMI and multivessel disease (MVD) in a limitedresource setting.

Dr Nauman Naseer, professor of cardiology at Akhtar Saeed Medical College and chief of cardiology at Bahria International Hospital, was the protagonist for complete revascularisation of significant N-IRA disease and Dr Ahmed Suliman, associate professor of cardiology at the University of Khartoum and cardiologist at the National Cardiothoracic Centre in Khartoum, Sudan, argued for infarct artery (IRA)-only revascularisation. The session was moderated by Dr Bernard Gersh, professor of medicine and consultant in the Department of Cardiovascular Diseases at the Mayo Clinic, College of Medicine, USA.

Both debaters agreed that the main focus is to achieve successful revascularisation of the IRA and the restoration of TIMI 3 flow but cited the evidence demonstrating that concomitant multivessel disease is frequently encountered in ST-elevation myocardial infarction (STEMI) patients and is associated with poorer outcomes than those who present with IRA disease only.

Prof Naseer explained the four possible strategies to deal with N-IRA significant disease: intervention within the setting of primary percutaneous intervention; a staged procedure during the index admission and before discharge; a staged procedure after discharge; or treating the N-IRA disease as per stable angina pectoris guidelines. Prof Naseer supported full revascularisation preferably during the same hospital admission, an approach endorsed by the ESC guidelines (class IIa indication).1 His main arguments were:

- Several randomised trials demonstrated the superiority of complete revascularisation of N-IRA versus IRA only. The largest four trials were PRAMI,^2^ CvLPRIT,^3^ DANAMI-3 PriMULTI^4^ and COMPARE-ACUTE5 trial (Table 1). Benefit was driven primarily by the need for repeat revascularisation with only a trend towards a reduction in hard end-points.
- FFR/iFR (fractional flow reserve/instantaneous free-wave ratio) helps to identify haemodynamically significant disease in non-culprit vessels.
- Many patients in the developing world live far away from cities and the need for urgent repeat revascularisation may translate into a mortality difference due to treatment delay.
- Saving costs initially via the IRA-only approach could incur higher costs at a later stage and this is particularly relevant in the African setting since most patients are liable for ‘out-of pocket’ expenses.

**Table 1:** Summary of outcomes of major trials comparing complete revascularisation versus IRA-only

*Study*	*Primary outcome*	*HR ( 95% CI)*	*p-value*	*Secondary outcomes*	*HR ( 95% CI)*	*p-value*
PRAMI	CV death, non-fatal MI and refractory angina	0.35 (0.21–0.58)	< 0.001	CV death	0.34 (0.11–1.08)	0.07
				Non-fatal MI	0.32 (0.13–0.75)	0.009
				Repeat revascularisation	0.30 ( 0.17–0.56)	< 0.001
CvLRIT	Death, non-fatal MI, heart failure, repeat revascularisation	0.45 (0.24–0.48)	0.009	Death	0.32 (0.06–1.60)	0.14
				Non-fatal MI	0.48 (0.09–2.62)	0.39
				Heart failure	0.43 (0.13–1.39)	0.14
				Repeat revascularisation	0.55 (0.22–1.39)	0.2
DANAMI-3 PriMULTI	Death, non-fatal MI and repeat revascularisation	0.56 (0.38–0.83)	0.004	Death	1.4 (0.63–3·0)	0.43
				Non-fatal MI	0.94 (0.47–1.9)	0.87
				Repeat revascularisation	0.31 (0.18–0.53)	< 0.001
COMPARE-ACUTE	Death, non-fatal MI, repeat revascularisation, CVA	0.35 (0.22–0.55)	< 0.001	Death	0.8 (0.25–2.56)	0.7
				Non-fatal MI	0.5 (0.22–1.13)	0.1
				Repeat Revascularisation	0.32 (0.20–0.54)	< 0.001

The counter argument that the initial strategy should be primary percutaneous coronary intervention (PPCI) of the IRA-only was made by Dr Suliman who presented the evidence cited below:

- All studies did not demonstrate a mortality benefit and event rates were primarily driven by the need for repeat revascularisation.
- All studies cited above performed complete revascularisation on the IRA-only without any attempt to risk-stratify the haemodynamic significance of the lesions in the non-IRA vessels using non-invasive testing. The Prague 13 trial, which excluded patients with angina more than a month before STEMI, was equivocal.
- Patients with left main stem or proximal significant disease in the N-IRA should undergo full revascularisation with PCI or coronary artery bypass grafting (CABG).
- FFR is not available in most cardiac catheterisation laboratories on the African continent.
- On average, one to two extra stents were used in patients undergoing complete revascularisation and extra contrast was used, adding a significant financial burden. Such valuable resources can be shifted to perform more primary angioplasty procedures for STEMI patients.

Dr Suliman concluded by advocating a strategy of culpritartery only and non-invasive ischaemia-testing for limited-resource settings. At the end of the debate, the audience was almost evenly split when polled regarding the two approaches. Results of ongoing trials are eagerly awaited to shed more light on the subject.
